# Relationship Between Severity of Comorbidities and Self-Perceptions of Aging

**DOI:** 10.1093/geroni/igab046.3731

**Published:** 2021-12-17

**Authors:** DaJung Chang

**Affiliations:** Miami University, Oxford, Ohio, United States

## Abstract

An older adult with negative self-perceptions of aging (SPA) can lead to lower self-rated health and a higher risk of mortality. To stay a positive SPA, keep a healthier status is very important. However, evidence also proved that health conditions, like a physical limitation, could predict the level of SPA. Older adults usually have a higher prevalence rate of chronic diseases than the younger population, which can adversely impact them. The purpose of this study is to determine the relationship between the severity of comorbidities and the change of SPA during a time. I examined data in 7,034 people from the 2012 wave Health and Retirement Study (HRS) and followed the respondents who have answer the SPA scale in the leave-behind questionnaire in 2016. The generalized estimating equation was used to analyze the relationship between the severity of comorbidities and SPA in different waves. To measure the severity of comorbidities, a reduced index of the comorbidities severity scale (CmSS) was created to collect the health condition from HRS. Results statistical model shows that an individual with more severity of comorbidities may have a more negative SPA. However, the relationship does not follow through with the times. These findings enhance the previous study that there are relationships between severity of comorbidities and SPA. The benefit of this study is to use a different measurement to identify the severity of comorbidities and extend to more in-depth research.

